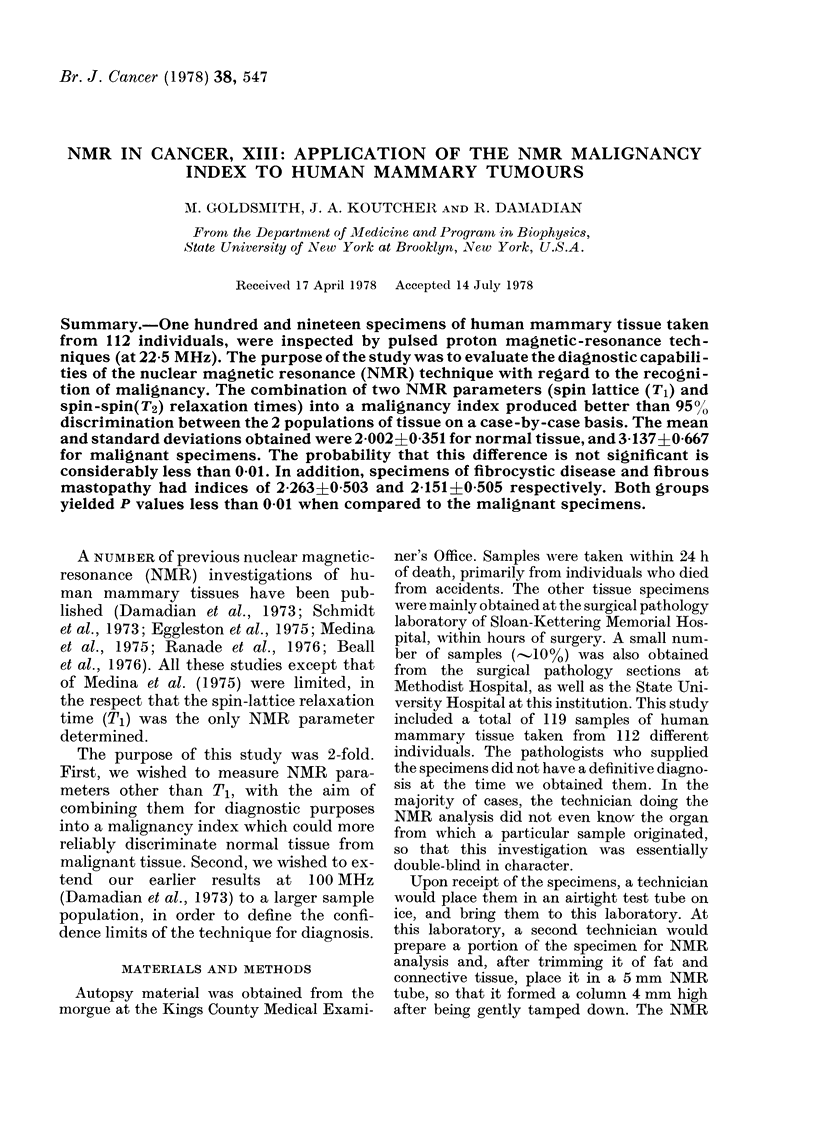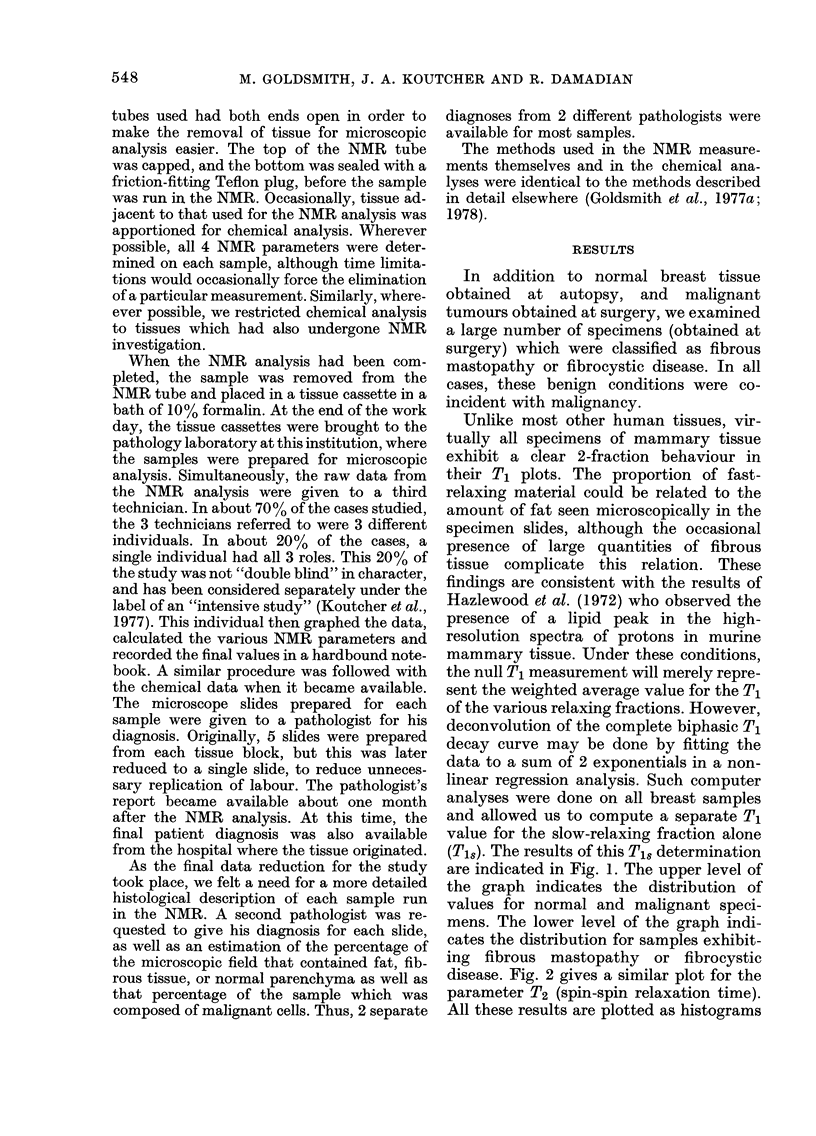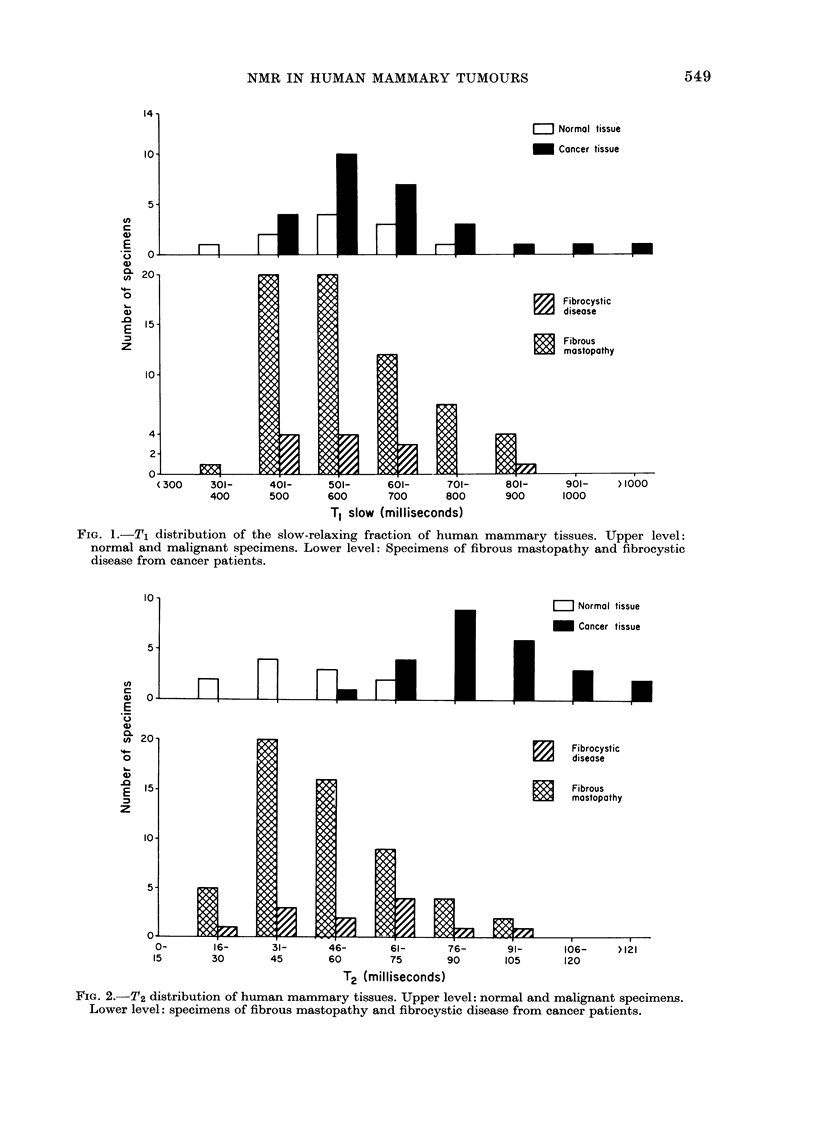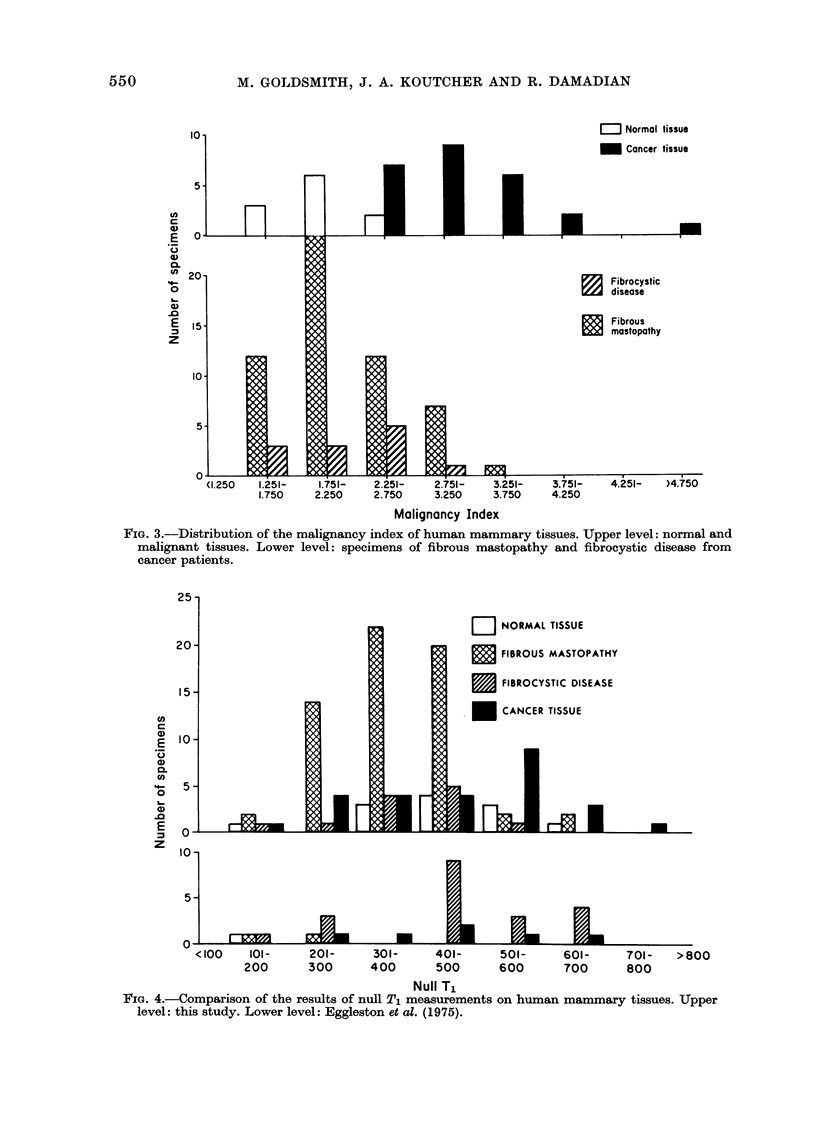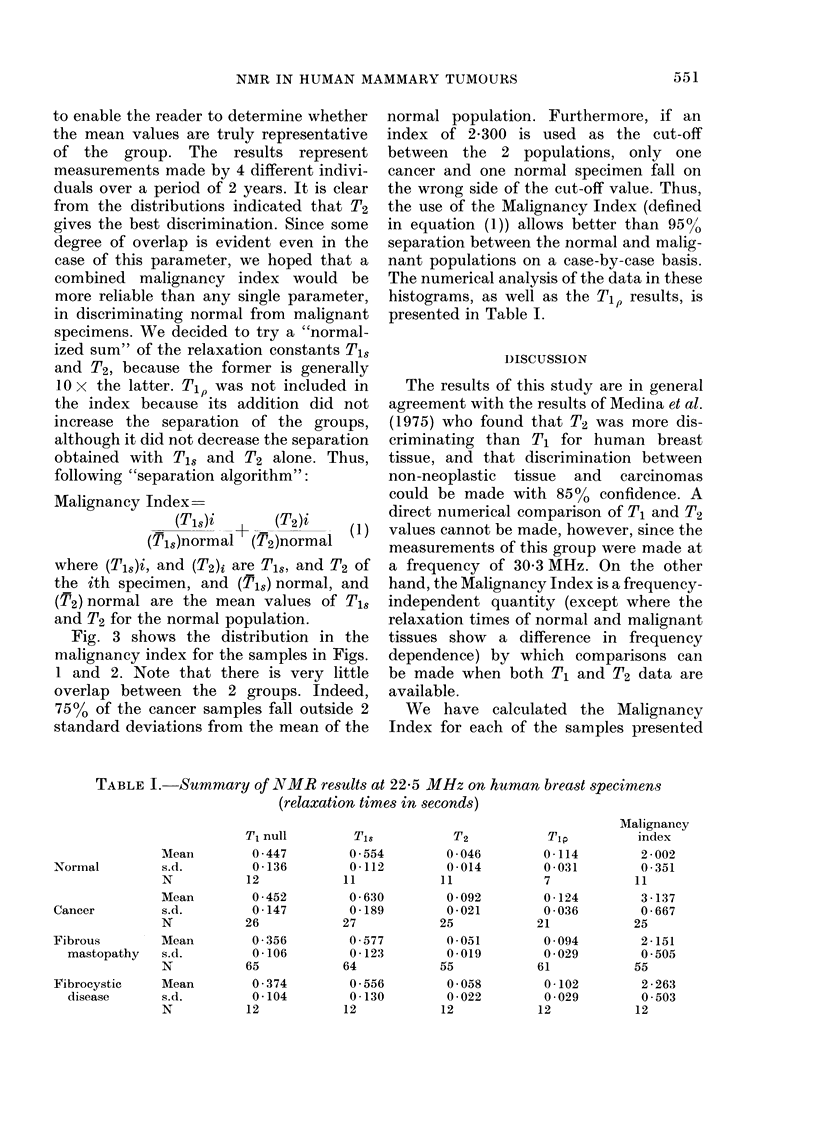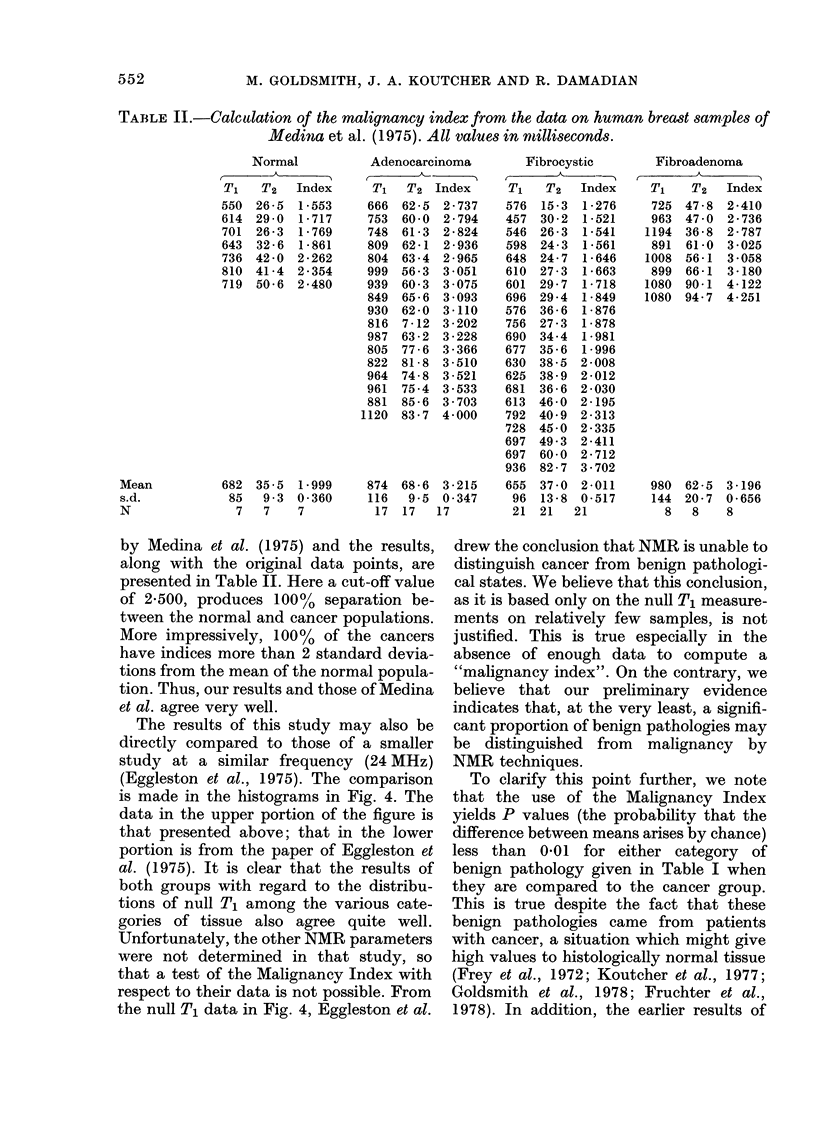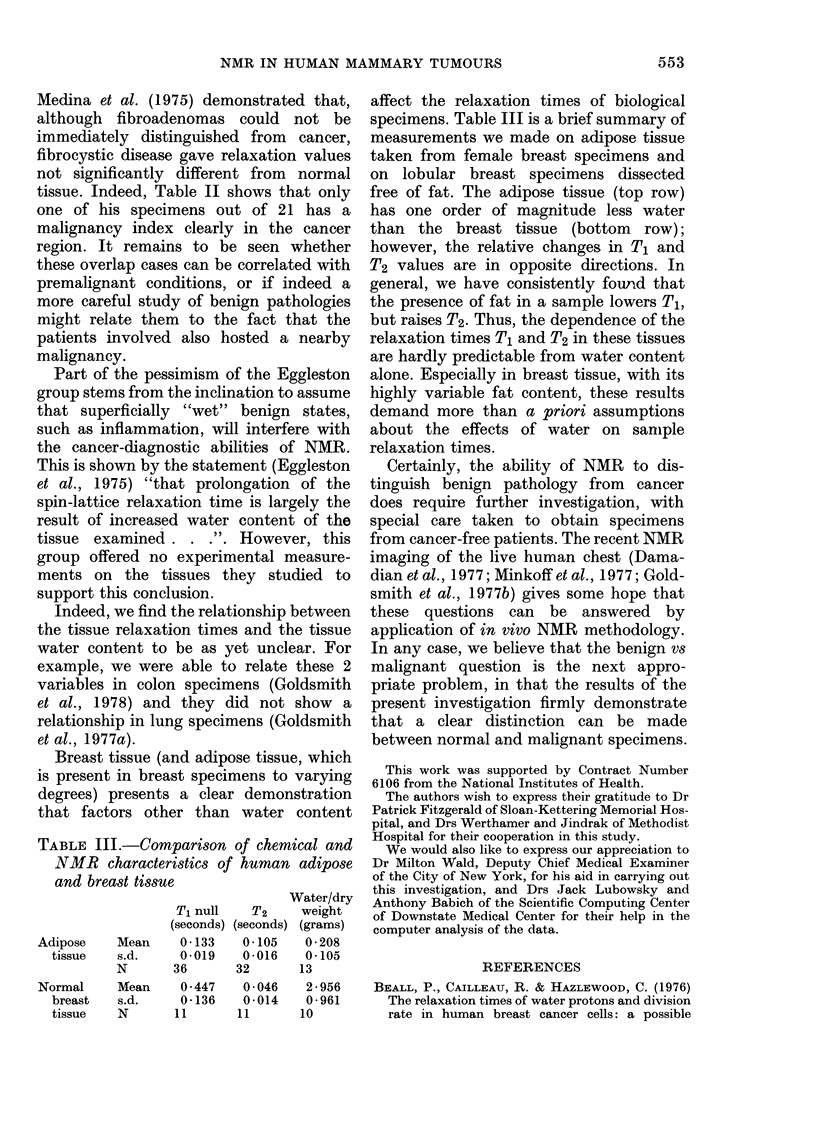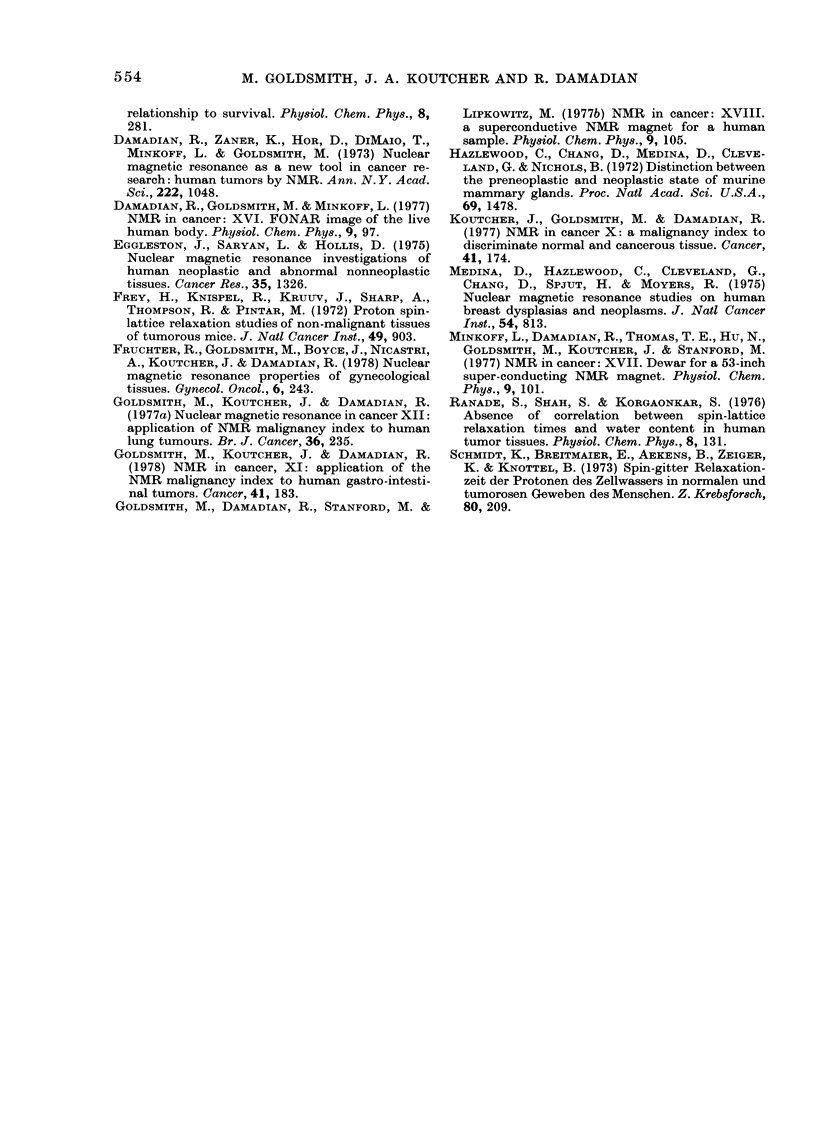# NMR in cancer, XIII: application of the NMR malignancy index to human mammary tumours.

**DOI:** 10.1038/bjc.1978.243

**Published:** 1978-10

**Authors:** M. Goldsmith, J. A. Koutcher, R. Damadian

## Abstract

One hundred and nineteen specimens of human mammary tissue taken from 112 individuals, were inspected by pulsed proton magnetic-resonance techniques (at 22.5 MH2). The purpose of the study was to evaluate the diagnostic capabilities of the nuclear magnetic resonance (NMR) technique with regard to the recognition of malignancy. The combination of two NMR parameters (spin lattice (T1) and spin-spin(T2) relaxation times) into a malignancy index produced better than 95% discrimination between the 2 populations of tissue on a case-by-case basis. The mean and standard deviations obtained were 2.002 +/- 0.351 for normal tissue, and 3.137 +/- 0.667 for malignant specimens. The probability that this difference is not significant is considerably less than 0.01. In addition, specimens of fibrocystic disease and fibrous mastopathy had indices of 2.263 +/- 0.503 and 2.151 +/- 0.505 respectively. Both groups yielded P values less than 0.01 when compared to the malignant specimens.


*Br. J. Cancer* (1978) **38**, 547

### NMR IN CANCER, XIII: APPLICATION OF THE NMR MALIGNANCY INDEX TO HUMAN MAMMARY TUMOURS

M. GOLDSMITH, J. A. KOUTCHER AND R. DAMADIAN

*From the Department of Medicine and Program in Biophysics, State University of New York at Brooklyn, New York, U.S.A.*

Received 17 April 1978 Accepted 14 July 1978

**Summary.—One hundred and nineteen specimens of human mammary tissue taken from 112 individuals, were inspected by pulsed proton magnetic-resonance techniques (at 22·5 MHz). The purpose of the study was to evaluate the diagnostic capabilities of the nuclear magnetic resonance (NMR) technique with regard to the recognition of malignancy. The combination of two NMR parameters (spin lattice ($T_1$) and spin-spin($T_2$) relaxation times) into a malignancy index produced better than 95% discrimination between the 2 populations of tissue on a case-by-case basis. The mean and standard deviations obtained were 2·002±0·351 for normal tissue, and 3·137±0·667 for malignant specimens. The probability that this difference is not significant is considerably less than 0·01. In addition, specimens of fibrocystic disease and fibrous mastopathy had indices of 2·263±0·503 and 2·151±0·505 respectively. Both groups yielded *P* values less than 0·01 when compared to the malignant specimens.**

A NUMBER of previous nuclear magnetic-resonance (NMR) investigations of human mammary tissues have been published (Damadian *et al.*, 1973; Schmidt *et al.*, 1973; Eggleston *et al.*, 1975; Medina *et al.*, 1975; Ranade *et al.*, 1976; Beall *et al.*, 1976). All these studies except that of Medina *et al.* (1975) were limited, in the respect that the spin-lattice relaxation time ($T_1$) was the only NMR parameter determined.

The purpose of this study was 2-fold. First, we wished to measure NMR parameters other than $T_1$, with the aim of combining them for diagnostic purposes into a malignancy index which could more reliably discriminate normal tissue from malignant tissue. Second, we wished to extend our earlier results at 100 MHz (Damadian *et al.*, 1973) to a larger sample population, in order to define the confidence limits of the technique for diagnosis.

### MATERIALS AND METHODS

Autopsy material was obtained from the morgue at the Kings County Medical Examiner's Office. Samples were taken within 24 h of death, primarily from individuals who died from accidents. The other tissue specimens were mainly obtained at the surgical pathology laboratory of Sloan-Kettering Memorial Hospital, within hours of surgery. A small number of samples (~10%) was also obtained from the surgical pathology sections at Methodist Hospital, as well as the State University Hospital at this institution. This study included a total of 119 samples of human mammary tissue taken from 112 different individuals. The pathologists who supplied the specimens did not have a definitive diagnosis at the time we obtained them. In the majority of cases, the technician doing the NMR analysis did not even know the organ from which a particular sample originated, so that this investigation was essentially double-blind in character.

Upon receipt of the specimens, a technician would place them in an airtight test tube on ice, and bring them to this laboratory. At this laboratory, a second technician would prepare a portion of the specimen for NMR analysis and, after trimming it of fat and connective tissue, place it in a 5 mm NMR tube, so that it formed a column 4 mm high after being gently tamped down. The NMR

tubes used had both ends open in order to make the removal of tissue for microscopic analysis easier. The top of the NMR tube was capped, and the bottom was sealed with a friction-fitting Teflon plug, before the sample was run in the NMR. Occasionally, tissue adjacent to that used for the NMR analysis was apportioned for chemical analysis. Wherever possible, all 4 NMR parameters were determined on each sample, although time limitations would occasionally force the elimination of a particular measurement. Similarly, where-ever possible, we restricted chemical analysis to tissues which had also undergone NMR investigation.

When the NMR analysis had been completed, the sample was removed from the NMR tube and placed in a tissue cassette in a bath of 10% formalin. At the end of the work day, the tissue cassettes were brought to the pathology laboratory at this institution, where the samples were prepared for microscopic analysis. Simultaneously, the raw data from the NMR analysis were given to a third technician. In about 70% of the cases studied, the 3 technicians referred to were 3 different individuals. In about 20% of the cases, a single individual had all 3 roles. This 20% of the study was not "double blind" in character, and has been considered separately under the label of an "intensive study" (Koutcher *et al.*, 1977). This individual then graphed the data, calculated the various NMR parameters and recorded the final values in a hardbound notebook. A similar procedure was followed with the chemical data when it became available. The microscope slides prepared for each sample were given to a pathologist for his diagnosis. Originally, 5 slides were prepared from each tissue block, but this was later reduced to a single slide, to reduce unnecessary replication of labour. The pathologist's report became available about one month after the NMR analysis. At this time, the final patient diagnosis was also available from the hospital where the tissue originated.

As the final data reduction for the study took place, we felt a need for a more detailed histological description of each sample run in the NMR. A second pathologist was requested to give his diagnosis for each slide, as well as an estimation of the percentage of the microscopic field that contained fat, fibrous tissue, or normal parenchyma as well as that percentage of the sample which was composed of malignant cells. Thus, 2 separate diagnoses from 2 different pathologists were available for most samples.

The methods used in the NMR measurements themselves and in the chemical analyses were identical to the methods described in detail elsewhere (Goldsmith *et al.*, 1977*a*; 1978).

### RESULTS

In addition to normal breast tissue obtained at autopsy, and malignant tumours obtained at surgery, we examined a large number of specimens (obtained at surgery) which were classified as fibrous mastopathy or fibrocystic disease. In all cases, these benign conditions were coincident with malignancy.

Unlike most other human tissues, virtually all specimens of mammary tissue exhibit a clear 2-fraction behaviour in their $T_1$ plots. The proportion of fast-relaxing material could be related to the amount of fat seen microscopically in the specimen slides, although the occasional presence of large quantities of fibrous tissue complicate this relation. These findings are consistent with the results of Hazlewood *et al.* (1972) who observed the presence of a lipid peak in the high-resolution spectra of protons in murine mammary tissue. Under these conditions, the null $T_1$ measurement will merely represent the weighted average value for the $T_1$ of the various relaxing fractions. However, deconvolution of the complete biphasic $T_1$ decay curve may be done by fitting the data to a sum of 2 exponentials in a non-linear regression analysis. Such computer analyses were done on all breast samples and allowed us to compute a separate $T_1$ value for the slow-relaxing fraction alone ($T_{1s}$). The results of this $T_{1s}$ determination are indicated in Fig. 1. The upper level of the graph indicates the distribution of values for normal and malignant specimens. The lower level of the graph indicates the distribution for samples exhibiting fibrous mastopathy or fibrocystic disease. Fig. 2 gives a similar plot for the parameter $T_2$ (spin-spin relaxation time). All these results are plotted as histograms

FIG. 1.—$T_1$ distribution of the slow-relaxing fraction of human mammary tissues. Upper level: normal and malignant specimens. Lower level: Specimens of fibrous mastopathy and fibrocystic disease from cancer patients.

FIG. 2.—$T_2$ distribution of human mammary tissues. Upper level: normal and malignant specimens. Lower level: specimens of fibrous mastopathy and fibrocystic disease from cancer patients.

FIG. 3.—Distribution of the malignancy index of human mammary tissues. Upper level: normal and malignant tissues. Lower level: specimens of fibrous mastopathy and fibrocystic disease from cancer patients.

FIG. 4.—Comparison of the results of null $T_1$ measurements on human mammary tissues. Upper level: this study. Lower level: Eggleston *et al.* (1975).

to enable the reader to determine whether the mean values are truly representative of the group. The results represent measurements made by 4 different individuals over a period of 2 years. It is clear from the distributions indicated that $T_2$ gives the best discrimination. Since some degree of overlap is evident even in the case of this parameter, we hoped that a combined malignancy index would be more reliable than any single parameter, in discriminating normal from malignant specimens. We decided to try a "normalized sum" of the relaxation constants $T_{1s}$ and $T_2$, because the former is generally $10\times$ the latter. $T_{1\rho}$ was not included in the index because its addition did not increase the separation of the groups, although it did not decrease the separation obtained with $T_{1s}$ and $T_2$ alone. Thus, following "separation algorithm":

$$\text{Malignancy Index} = \frac{(T_{1s})i}{(\bar{T}_{1s})\text{normal}} + \frac{(T_2)i}{(\bar{T}_2)\text{normal}} \quad (1)$$

where $(T_{1s})i$, and $(T_2)_i$ are $T_{1s}$, and $T_2$ of the $i$th specimen, and $(\bar{T}_{1s})$ normal, and $(\bar{T}_2)$ normal are the mean values of $T_{1s}$ and $T_2$ for the normal population.

Fig. 3 shows the distribution in the malignancy index for the samples in Figs. 1 and 2. Note that there is very little overlap between the 2 groups. Indeed, 75% of the cancer samples fall outside 2 standard deviations from the mean of the normal population. Furthermore, if an index of 2·300 is used as the cut-off between the 2 populations, only one cancer and one normal specimen fall on the wrong side of the cut-off value. Thus, the use of the Malignancy Index (defined in equation (1)) allows better than 95% separation between the normal and malignant populations on a case-by-case basis. The numerical analysis of the data in these histograms, as well as the $T_{1\rho}$ results, is presented in Table I.

### DISCUSSION

The results of this study are in general agreement with the results of Medina *et al.* (1975) who found that $T_2$ was more discriminating than $T_1$ for human breast tissue, and that discrimination between non-neoplastic tissue and carcinomas could be made with 85% confidence. A direct numerical comparison of $T_1$ and $T_2$ values cannot be made, however, since the measurements of this group were made at a frequency of 30·3 MHz. On the other hand, the Malignancy Index is a frequency-independent quantity (except where the relaxation times of normal and malignant tissues show a difference in frequency dependence) by which comparisons can be made when both $T_1$ and $T_2$ data are available.

We have calculated the Malignancy Index for each of the samples presented

TABLE I.—*Summary of NMR results at 22·5 MHz on human breast specimens (relaxation times in seconds)*

| | | $T_1$ null | $T_{1s}$ | $T_2$ | $T_{1\rho}$ | Malignancy index |
|---|---|---|---|---|---|---|
| | Mean | 0·447 | 0·554 | 0·046 | 0·114 | 2·002 |
| Normal | s.d. | 0·136 | 0·112 | 0·014 | 0·031 | 0·351 |
| | N | 12 | 11 | 11 | 7 | 11 |
| | Mean | 0·452 | 0·630 | 0·092 | 0·124 | 3·137 |
| Cancer | s.d. | 0·147 | 0·189 | 0·021 | 0·036 | 0·667 |
| | N | 26 | 27 | 25 | 21 | 25 |
| Fibrous | Mean | 0·356 | 0·577 | 0·051 | 0·094 | 2·151 |
| mastopathy | s.d. | 0·106 | 0·123 | 0·019 | 0·029 | 0·505 |
| | N | 65 | 64 | 55 | 61 | 55 |
| Fibrocystic | Mean | 0·374 | 0·556 | 0·058 | 0·102 | 2·263 |
| disease | s.d. | 0·104 | 0·130 | 0·022 | 0·029 | 0·503 |
| | N | 12 | 12 | 12 | 12 | 12 |

TABLE II.—*Calculation of the malignancy index from the data on human breast samples of Medina* et al. (1975). *All values in milliseconds.*

| | Normal | | | Adenocarcinoma | | | Fibrocystic | | | Fibroadenoma | | |
|---|---|---|---|---|---|---|---|---|---|---|---|---|
| | $T_1$ | $T_2$ | Index | $T_1$ | $T_2$ | Index | $T_1$ | $T_2$ | Index | $T_1$ | $T_2$ | Index |
| | 550 | 26·5 | 1·553 | 666 | 62·5 | 2·737 | 576 | 15·3 | 1·276 | 725 | 47·8 | 2·410 |
| | 614 | 29·0 | 1·717 | 753 | 60·0 | 2·794 | 457 | 30·2 | 1·521 | 963 | 47·0 | 2·736 |
| | 701 | 26·3 | 1·769 | 748 | 61·3 | 2·824 | 546 | 26·3 | 1·541 | 1194 | 36·8 | 2·787 |
| | 643 | 32·6 | 1·861 | 809 | 62·1 | 2·936 | 598 | 24·3 | 1·561 | 891 | 61·0 | 3·025 |
| | 736 | 42·0 | 2·262 | 804 | 63·4 | 2·965 | 648 | 24·7 | 1·646 | 1008 | 56·1 | 3·058 |
| | 810 | 41·4 | 2·354 | 999 | 56·3 | 3·051 | 610 | 27·3 | 1·663 | 899 | 66·1 | 3·180 |
| | 719 | 50·6 | 2·480 | 939 | 60·3 | 3·075 | 601 | 29·7 | 1·718 | 1080 | 90·1 | 4·122 |
| | | | | 849 | 65·6 | 3·093 | 696 | 29·4 | 1·849 | 1080 | 94·7 | 4·251 |
| | | | | 930 | 62·0 | 3·110 | 576 | 36·6 | 1·876 | | | |
| | | | | 816 | 7·12 | 3·202 | 756 | 27·3 | 1·878 | | | |
| | | | | 987 | 63·2 | 3·228 | 690 | 34·4 | 1·981 | | | |
| | | | | 805 | 77·6 | 3·366 | 677 | 35·6 | 1·996 | | | |
| | | | | 822 | 81·8 | 3·510 | 630 | 38·5 | 2·008 | | | |
| | | | | 964 | 74·8 | 3·521 | 625 | 38·9 | 2·012 | | | |
| | | | | 961 | 75·4 | 3·533 | 681 | 36·6 | 2·030 | | | |
| | | | | 881 | 85·6 | 3·703 | 613 | 46·0 | 2·195 | | | |
| | | | | 1120 | 83·7 | 4·000 | 792 | 40·9 | 2·313 | | | |
| | | | | | | | 728 | 45·0 | 2·335 | | | |
| | | | | | | | 697 | 49·3 | 2·411 | | | |
| | | | | | | | 697 | 60·0 | 2·712 | | | |
| | | | | | | | 936 | 82·7 | 3·702 | | | |
| Mean | 682 | 35·5 | 1·999 | 874 | 68·6 | 3·215 | 655 | 37·0 | 2·011 | 980 | 62·5 | 3·196 |
| s.d. | 85 | 9·3 | 0·360 | 116 | 9·5 | 0·347 | 96 | 13·8 | 0·517 | 144 | 20·7 | 0·656 |
| N | 7 | 7 | 7 | 17 | 17 | 17 | 21 | 21 | 21 | 8 | 8 | 8 |

by Medina *et al.* (1975) and the results, along with the original data points, are presented in Table II. Here a cut-off value of 2·500, produces 100% separation between the normal and cancer populations. More impressively, 100% of the cancers have indices more than 2 standard deviations from the mean of the normal population. Thus, our results and those of Medina *et al.* agree very well.

The results of this study may also be directly compared to those of a smaller study at a similar frequency (24 MHz) (Eggleston *et al.*, 1975). The comparison is made in the histograms in Fig. 4. The data in the upper portion of the figure is that presented above; that in the lower portion is from the paper of Eggleston *et al.* (1975). It is clear that the results of both groups with regard to the distributions of null $T_1$ among the various categories of tissue also agree quite well. Unfortunately, the other NMR parameters were not determined in that study, so that a test of the Malignancy Index with respect to their data is not possible. From the null $T_1$ data in Fig. 4, Eggleston *et al.* drew the conclusion that NMR is unable to distinguish cancer from benign pathological states. We believe that this conclusion, as it is based only on the null $T_1$ measurements on relatively few samples, is not justified. This is true especially in the absence of enough data to compute a "malignancy index". On the contrary, we believe that our preliminary evidence indicates that, at the very least, a significant proportion of benign pathologies may be distinguished from malignancy by NMR techniques.

To clarify this point further, we note that the use of the Malignancy Index yields $P$ values (the probability that the difference between means arises by chance) less than 0·01 for either category of benign pathology given in Table I when they are compared to the cancer group. This is true despite the fact that these benign pathologies came from patients with cancer, a situation which might give high values to histologically normal tissue (Frey *et al.*, 1972; Koutcher *et al.*, 1977; Goldsmith *et al.*, 1978; Fruchter *et al.*, 1978). In addition, the earlier results of

Medina *et al.* (1975) demonstrated that, although fibroadenomas could not be immediately distinguished from cancer, fibrocystic disease gave relaxation values not significantly different from normal tissue. Indeed, Table II shows that only one of his specimens out of 21 has a malignancy index clearly in the cancer region. It remains to be seen whether these overlap cases can be correlated with premalignant conditions, or if indeed a more careful study of benign pathologies might relate them to the fact that the patients involved also hosted a nearby malignancy.

Part of the pessimism of the Eggleston group stems from the inclination to assume that superficially "wet" benign states, such as inflammation, will interfere with the cancer-diagnostic abilities of NMR. This is shown by the statement (Eggleston *et al.*, 1975) "that prolongation of the spin-lattice relaxation time is largely the result of increased water content of the tissue examined . . .". However, this group offered no experimental measurements on the tissues they studied to support this conclusion.

Indeed, we find the relationship between the tissue relaxation times and the tissue water content to be as yet unclear. For example, we were able to relate these 2 variables in colon specimens (Goldsmith *et al.*, 1978) and they did not show a relationship in lung specimens (Goldsmith *et al.*, 1977*a*).

Breast tissue (and adipose tissue, which is present in breast specimens to varying degrees) presents a clear demonstration that factors other than water content affect the relaxation times of biological specimens. Table III is a brief summary of measurements we made on adipose tissue taken from female breast specimens and on lobular breast specimens dissected free of fat. The adipose tissue (top row) has one order of magnitude less water than the breast tissue (bottom row); however, the relative changes in $T_1$ and $T_2$ values are in opposite directions. In general, we have consistently found that the presence of fat in a sample lowers $T_1$, but raises $T_2$. Thus, the dependence of the relaxation times $T_1$ and $T_2$ in these tissues are hardly predictable from water content alone. Especially in breast tissue, with its highly variable fat content, these results demand more than *a priori* assumptions about the effects of water on sample relaxation times.

TABLE III.—*Comparison of chemical and NMR characteristics of human adipose and breast tissue*

| | | $T_1$ null (seconds) | $T_2$ (seconds) | Water/dry weight (grams) |
|---|---|---|---|---|
| Adipose tissue | Mean | 0·133 | 0·105 | 0·208 |
| | s.d. | 0·019 | 0·016 | 0·105 |
| | N | 36 | 32 | 13 |
| Normal breast tissue | Mean | 0·447 | 0·046 | 2·956 |
| | s.d. | 0·136 | 0·014 | 0·961 |
| | N | 11 | 11 | 10 |

Certainly, the ability of NMR to distinguish benign pathology from cancer does require further investigation, with special care taken to obtain specimens from cancer-free patients. The recent NMR imaging of the live human chest (Damadian *et al.*, 1977; Minkoff *et al.*, 1977; Goldsmith *et al.*, 1977*b*) gives some hope that these questions can be answered by application of *in vivo* NMR methodology. In any case, we believe that the benign *vs* malignant question is the next appropriate problem, in that the results of the present investigation firmly demonstrate that a clear distinction can be made between normal and malignant specimens.

This work was supported by Contract Number 6106 from the National Institutes of Health.

The authors wish to express their gratitude to Dr Patrick Fitzgerald of Sloan-Kettering Memorial Hospital, and Drs Werthamer and Jindrak of Methodist Hospital for their cooperation in this study.

We would also like to express our appreciation to Dr Milton Wald, Deputy Chief Medical Examiner of the City of New York, for his aid in carrying out this investigation, and Drs Jack Lubowsky and Anthony Babich of the Scientific Computing Center of Downstate Medical Center for their help in the computer analysis of the data.

### REFERENCES

BEALL, P., CAILLEAU, R. & HAZLEWOOD, C. (1976) The relaxation times of water protons and division rate in human breast cancer cells: a possible relationship to survival. *Physiol. Chem. Phys.*, **8**, 281.

DAMADIAN, R., ZANER, K., HOR, D., DIMAIO, T., MINKOFF, L. & GOLDSMITH, M. (1973) Nuclear magnetic resonance as a new tool in cancer research: human tumors by NMR. *Ann. N.Y. Acad. Sci.*, **222**, 1048.

DAMADIAN, R., GOLDSMITH, M. & MINKOFF, L. (1977) NMR in cancer: XVI. FONAR image of the live human body. *Physiol. Chem. Phys.*, **9**, 97.

EGGLESTON, J., SARYAN, L. & HOLLIS, D. (1975) Nuclear magnetic resonance investigations of human neoplastic and abnormal nonneoplastic tissues. *Cancer Res.*, **35**, 1326.

FREY, H., KNISPEL, R., KRUUV, J., SHARP, A., THOMPSON, R. & PINTAR, M. (1972) Proton spin-lattice relaxation studies of non-malignant tissues of tumorous mice. *J. Natl Cancer Inst.*, **49**, 903.

FRUCHTER, R., GOLDSMITH, M., BOYCE, J., NICASTRI, A., KOUTCHER, J. & DAMADIAN, R. (1978) Nuclear magnetic resonance properties of gynecological tissues. *Gynecol. Oncol.*, **6**, 243.

GOLDSMITH, M., KOUTCHER, J. & DAMADIAN, R. (1977*a*) Nuclear magnetic resonance in cancer XII: application of NMR malignancy index to human lung tumours. *Br. J. Cancer*, **36**, 235.

GOLDSMITH, M., KOUTCHER, J. & DAMADIAN, R. (1978) NMR in cancer, XI: application of the NMR malignancy index to human gastro-intestinal tumors. *Cancer*, **41**, 183.

GOLDSMITH, M., DAMADIAN, R., STANFORD, M. & LIPKOWITZ, M. (1977*b*) NMR in cancer: XVIII. a superconductive NMR magnet for a human sample. *Physiol. Chem. Phys.*, **9**, 105.

HAZLEWOOD, C., CHANG, D., MEDINA, D., CLEVELAND, G. & NICHOLS, B. (1972) Distinction between the preneoplastic and neoplastic state of murine mammary glands. *Proc. Natl Acad. Sci. U.S.A.*, **69**, 1478.

KOUTCHER, J., GOLDSMITH, M. & DAMADIAN, R. (1977) NMR in cancer X: a malignancy index to discriminate normal and cancerous tissue. *Cancer*, **41**, 174.

MEDINA, D., HAZLEWOOD, C., CLEVELAND, G., CHANG, D., SPJUT, H. & MOYERS, R. (1975) Nuclear magnetic resonance studies on human breast dysplasias and neoplasms. *J. Natl Cancer Inst.*, **54**, 813.

MINKOFF, L., DAMADIAN, R., THOMAS, T. E., HU, N., GOLDSMITH, M., KOUTCHER, J. & STANFORD, M. (1977) NMR in cancer: XVII. Dewar for a 53-inch super-conducting NMR magnet. *Physiol. Chem. Phys.*, **9**, 101.

RANADE, S., SHAH, S. & KORGAONKAR, S. (1976) Absence of correlation between spin-lattice relaxation times and water content in human tumor tissues. *Physiol. Chem. Phys.*, **8**, 131.

SCHMIDT, K., BREITMAIER, E., AEKENS, B., ZEIGER, K. & KNOTTEL, B. (1973) Spin-gitter Relaxationzeit der Protonen des Zellwassers in normalen und tumorosen Geweben des Menschen. *Z. Krebsforsch*, **80**, 209.